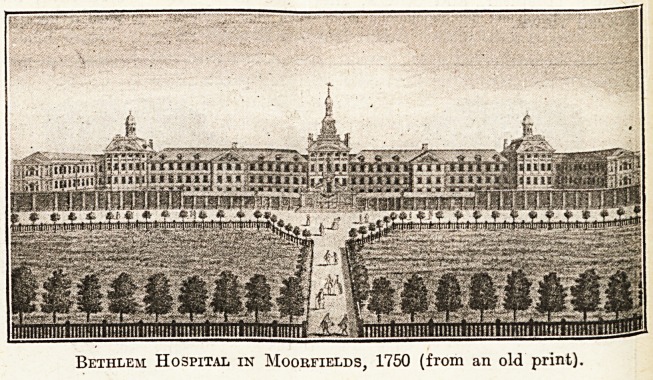# As Depicted by Engraved Views.—II

**Published:** 1920-12-04

**Authors:** 


### THE HISTORY OF HOSPITALS

### AS DEPICTED BY ENGRAVED VIEWS.—II.

Of hospitals which are hostels and not spittals, such as the Foundling Hospital, Christ's Hospital, Aske's Hospital, and others, there are many engraved views.

The Foundling Hospital, 1752 (from an old print).

The Foundling Hospital has been drawn, painted, and engraved many times, and a large collection of prints can be made with inconsiderable trouble and at moderate expense. And as a record of bygone days the value of the collection will be enhanced should the institution, as is probable, take up its future home in the country, where it is thought that the children will be happier and better than in the centre of a great city. At the date of the view of the front of the building (about 1752) that is reproduced on this page, the institution was virtually in the country, and in the middle of the large estate which the Governors were obliged to buy entire (fortunately, as it turned out) from the Earl of Salisbury. The purchase price was £7,000, of which £500 was returned as a donation. Marshall's statue of Captain Coram, the Founder, which now stands on the pedestal between the two entrance gates, was not erected until 100 years after the date of our illustration.

A Possible University Site.

There are many reasons why the Foundling estate should have been considered eminently suitable as a new site for the University of London. It possesses a considerable acreage of ground, unbroken by streets, for the main buildings; moreover, surrounding this site are large blocks of leasehold property belonging to the institution which can quickly be made use of in future developments. Moreover, the present structure, largely used for educational purposes, would be quite serviceable as a home for the University while the considerable time elapses during which new buildings can be erected. Our view indicates to some extent the land immediately available for building purposes.

Our other illustration is of the chapel. It is taken from an aquatint in colours in one of the early nineteenth-century books published by Ackermann, from which single plates may ocasionally be bought from dealers. The architectural drawing is by Pugin, and the figures by Rowlandson. The chapel organ was presented by Handel, who was present at its opening and subsequently gave many performances of the "Messiah" for the benefit of the charity.

The original building was after the design of Theodore Jacobsen, who was also architect of the Royal Hospital at Gosport. Mr. Henry Currey, the architect of St. Thomas's Hospital, was responsible for the plans of some of the nineteenth-century additions. The whole of the buildings are of fine proportion, and their artistic contents are very remarkable, including pictures by Hogarth, Reynolds, and other great artists. In the topographical interest special mention may be made of a set of circular panels on the walls of the Court

The Foundling Chapel, 1801 (from an old print).

Room, depicting Greenwich Hospital, Christ's Hospital, and St. Thomas's Hospital, by Samuel Wall, R.A.; Chelsea and Bethlem Hospitals, by Haytley; the Charterhouse, by the great Gainsborough; and St. George's and the Foundling Hospitals, by G. R. Wilson, R.A.

### Old Bethlem Hospital.

Old Bethlem Hospital was a few weeks ago much before the public on the occasion of the opening of a new and complete *x*-ray department. Bethlem is a very up-to-date institution; here the students of St. Thomas's, University College, Guy's, London, and the Royal Free Hospitals attend for instruction in an important branch of medicine, and by means of the developments of recent years the sister sciences of neurology and psychiatry flourish side by side. The possibilities of the use of *x*-rays in research and treatment promise much for the hopeful and curable side of the cases.

Bethlem Hospital in Moorfields, 1750 (from an old print).

Our illustration is of the old Bedlam about the year 1750, then situated in "Moore Fields," whence also has departed the Royal London Ophthalmic Hospital to the City Road. The physicians of the old hospital would rub their eyes if they could come to life and see the everyday work at the newer Bethlem Hospital, erected in St. George's Fields, now Lambeth Road, in 1815. The old hospital is the subject of an illustration in Stow's "Survey," under which is the following inscription:

Bethlem Hospital, Lambeth, 1815 (from an old print).

This Hospital was first founded in the Year 1246, to be a Priory of Canons and Sisters to receive the Bishop of Bethem, &c., when he should have occasion to travel hither. K. Henry 8th gave the House to the Citizens of London, and they with great Charge converted it into an Hospital for Lunaticks. It formerly stood in an obscure place, and was to little and close to receive the number of Patients brought thither, but 'tis now erected against the City Wall, fronting the Fields, in a very pleasant Airy Situation. It is a commodious and spacious Structure, built of Brick and Free Stone, handsomely Adorn'd with Ornaments and Carving &c. it is 540 foot long, and 40 broad besides the Wall which incloseth the Gardens before it and contains Appartments for 150 Lunaticks, 60 or 70 being generally cured there every Year.

### Bethlem in Romance.

Lovers of William de Morgan's books will remember how in "Alice-for-Short" the poor painter Verrinder lived in the attic of a high building in South London, from the window of which a visitor noticed a dome appearing over the housetops. The painter said in answer to a question: "The asylum? Yes. Bedlam, if you want to know." He did not add that his wife had been beneath this dome for fifty years and that his one hold on life was his daily look at it. That would not be told in so little oblique a manner in a de Morgan novel!

### Decompression at Ninety.

The story is symbolical of the scientific progress of Bethlem. Fifty years before, the painter's wife had fallen downstairs and had remained unconscious and spoon-fed ever since. Why she went to Bethlem we cannot say, but we find her at ninety an object of interest to a mental specialist who comes across the patient by chance. Says he: "I was going to say that they had a consultation over the case at my suggestion, and they won't have it—except Paisley—that she is a case of traumatic insanity. . . . And I believe there would be a possibility—it's only a possibility, mind—that if she were trepanned some mental revival might take place."

Decompression performed on an old lady of ninety is no joke, but she had it—it was necessary to the evolution of the story—and lived happily ever after, a dear old thing who was able in a marvellous way to clear up obscure matters and put Alice-for-Short in the way of getting hold of her fortune.

The previous article appeared on November 13, p. 141.

## Figures and Tables

**Figure f1:**
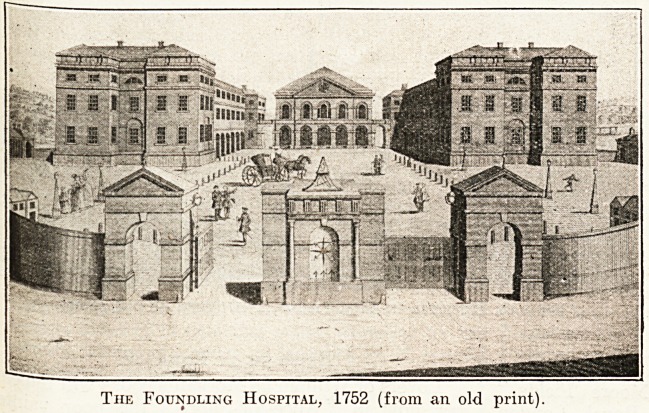


**Figure f2:**
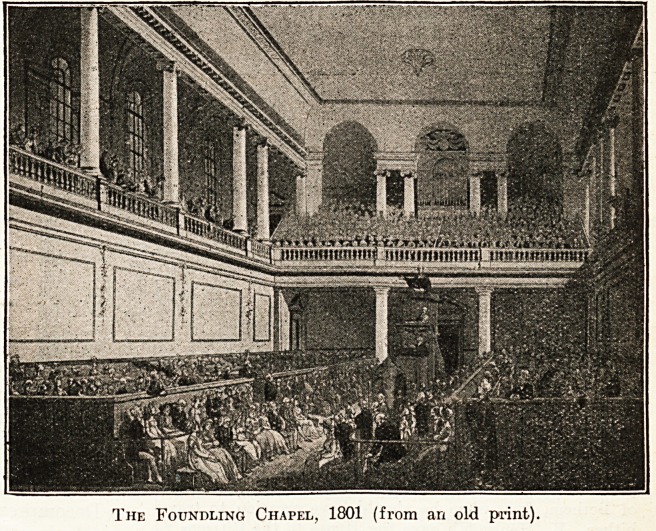


**Figure f3:**
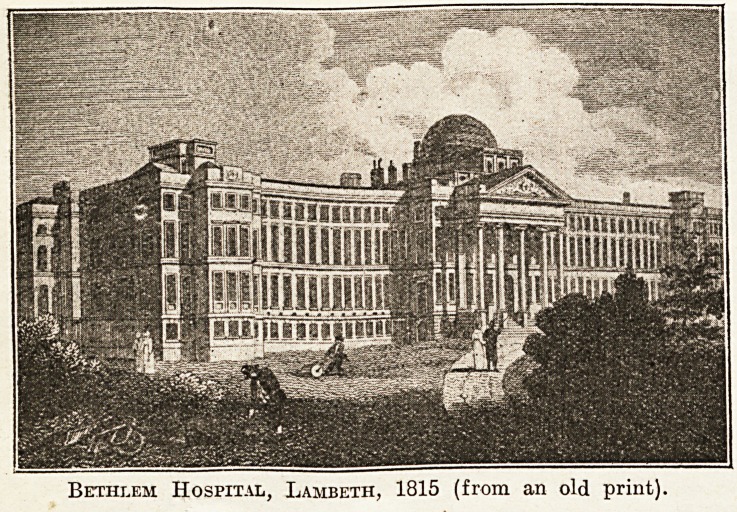


**Figure f4:**